# New oncogenic functions of LINE1 retroelement as a ceRNA for tumor suppressive microRNA miR-126 on ENPP5

**DOI:** 10.1371/journal.pone.0286814

**Published:** 2023-06-23

**Authors:** Kang-Hoon Lee, Hyeon-Ji Hwang, Yeo-Jin Im, A-Reum Nam, Jeong-Woon Lee, Je-Yoel Cho

**Affiliations:** 1 Department of Biochemistry, BK21 Plus and Research Institute for Veterinary Science, School of Veterinary Medicine, Seoul National University, Seoul, Korea; 2 Comparative Medicine Disease Research Center, Seoul National University, Seoul, Republic of Korea; Royan Institute for Stem Cell Biology and Technology, ISLAMIC REPUBLIC OF IRAN

## Abstract

Retroelements (REs) had been considered ’Junk’ until the encyclopedia of DNA elements (ENCODE) project demonstrated that most genome is functional. Although the function of retroelements has been reported in diverse cancers including human breast cancer (HBC) and subtypes, only a few studies have suggested the putative functions of REs via their random genome integration. A canine mammary tumor (CMT) has been highlighted due to the similarities in molecular and pathophysiology with HBC. This study investigated the putative roles of REs common in both HBC and CMT. The human LINE and HERV-K sequences harbor many miRNAs responsive elements (MREs) for tumor-suppressive miRNA such as let-7. We also observed that various MREs are exist in the ERV and LINE highly expressed in the transcriptome data of CMT as well as HBC sets. MREs against miR-126 were highly expressed in both HBC and CMT while the levels of miR-126 were down-regulated. Oppositely, the expression of miR-126 target genes was significantly up-regulated in the cancers. Moreover, cancer patients with an increased level of miR-126 showed better overall survival. The expression of ENPP5, a putative miR-126 target gene, was downregulated by miR-126 mimic. Importantly, overexpression of LINE fragment significantly suppressed miR-126 function on the target gene expression. We propose the functional role of REs expression in tumorigenesis as competing endogenous RNAs (ceRNA) against tumor-suppressive miRNAs. This study provided pieces of evidence that LINE expression, even partial and fragmented, have a regulatory function in ENPP5 gene expression via the competition with miR-126.

## Introduction

It is not surprising any more that almost half of our genome is composed of retroelements (also called retrotransposons; REs) which has been known as ‘Junk DNA’ [1]. Around 42% of the human genome is made up of long-and short-interspersed nuclear elements (LINE, SINE; non-LTR (long terminal repeat) retrotransposons) and endogenous retrovirus (ERVs; LTR retrotransposons) when protein-coding genes consist of 1.2% of the human genome. ERVs and LINEs that consists about 8% and 20% of human genome respectively, are capable of autonomously replicating using own open reading frames that encode proteins for retro-transposition mechanisms involving the reverse transcription of RNA intermediates and the insertion of the resulting cDNA copies in the host genome. However, SINEs occupying 13% of human genome lack the retro-transposition machinery and depend on LINEs [2]. The vast majority of the RE’s content in genomes are still considered as defective copies of REs.

For the last few decades, the function of REs expressed in some disease conditions including cancers has been suggested with various mechanisms depending on transposable activity and protein coding potentials [3, 4]. Several oncogenic mechanisms of human endogenous retroviruses (HERVs) have been reported [5, 6]. For instance, enhancing host transcriptional machinery which cause uncontrolled cell proliferation by long terminal repeats (LTRs) structures of HERV was found in Hodgkin’s lymphoma [7]. On the other hand, oncogenic proteins such as Np9 and Rec encoded by HERV-K and the immune suppressive function of envelop protein can also promote tumor formation and development [8]. In addition, genome instability via the expansion of REs has been known as the major mechanism of REs in various cancers [9]. HERV-induced chromosomal translocation in somatic cells has been reported in prostate cancer and lung adenocarcinoma [10, 11]. It has also been demonstrated that REs including LINE, ALU and SVA randomly spread on genome and often insert themselves in genomic regions with crucial biological functions resulting in insertional mutagenesis [12]. Additionally, DNA double strand breaks generated by expression of LINE1 ORF2 has been suggested as a possible mechanism that LINE contributes to genome instability in cancers non-allelic homologous recombination is the major mechanism that involves Alu elements [13].

Of note, most of REs are found to be transcribed as non-coding RNAs (ncRNAs) with unexpected implications of novel genetic mechanisms in tumorigenesis [14, 15]. The encyclopedia of DNA elements (ENCODE) project demonstrated that the most genome is functional. Surprisingly, about 85% of the human genome is transcribed into primary and processed transcripts without coding potentials [16]. A microRNA (miRNA) is a small ncRNA that functions in RNA silencing and post-transcriptional regulation of gene expression [17]. Interestingly, intergenic RE sequences were suggested as molecular origins of many miRNAs. For instance, Piriyapongsa et al. evaluated the contribution of REs to human miRNAs and found that many human miRNAs were derived from REs (LINE and SINE, and LTRs) [18]. Hsa-miR-552, -616 and -548a-2 involved in tumorigenesis were identified as REs-derived miRNAs [19–21]. Moreover, many miRNAs are also evolutionarily conserved, which implies that they have important biological functions [22]. On the other hand, some regulatory mechanisms such as competing endogenous RNAs (ceRNAs) or miRNA sponge are recently suggested in the regulation of miRNA expression. CeRNAs that share miRNA responsive elements (MREs) recently entered the complex regulations of miRNA on gene expression [23]. Indeed, the better understanding in the crosstalk among REs-miRNAs-genes is crucial to delineate the complexity in gene regulation.

Dogs and canine mammary tumor (CMT) have been paid attention as a model for human breast cancers. Dogs develop spontaneous CMT with clinical and molecular similarities to human breast cancer (HBC) [24]. Some clinical factors such as onset age, hormonal etiology, tumor size, stage and lymph node invasion that are closely link to outcome, are also similar between HBC and CMT [25]. In addition, many HBC associated molecules such as steroid receptors, epidermal growth factor, proliferation markers, metalloproteinase and cyclooxygenase, and the mutation of tumor suppressor gene, p53 have been identified and characterized in CMT too [26]. These similarities in CMT to HBC indicate that the CMT could be an excellent model for the study of HBC despite of discrepancy in the usage of histological classification between CMT and HBC. For the first time, we revealed the new functional role of REs robustly increased in both HBC and CMT and suggest the axis of REs-tumor suppressive miRNA-target gene expressions as a crucial role of REs in the HBC and CMT oncogenesis.

## Materials and methods

### Data acquisition and identification of differential expressed retroelements

HBC and CMT transcriptome data was obtained from the Gene expresson Omnibus (GEO) of National Center for Biotechnology Information (NCBI) (Accession ID: GSE110114 and PRJNA527698). The information of interspersed repeats and low complexity DNA were downloaded from Repeatmasker database (ver.4.0.5) [27] and all repetitive sequence positions were extracted and converted into bed formatted data. After that, only the data of LINEs and LTRs, which are focused on this study, was filtered. To calculate the expression of LINEs and LTRs, the repetitive sequences overlapped with genes on same strand were removed to eliminate read count bias caused by overlapping genes. For this step, the analysis was carried out as follows: (1) Gene body regions were extracted from ENSEMBL annotation v91 and converted into bed format; (2) Filtering was performed using intersect function of Bedtools with options (-s -v). Total mapped reads were calculated from the alignment file of RNA-seq using the idxstats function of Samtools, and the mapped reads on the selected LINEs and LTRs were counted using the multicov function of Bedtools with options (-s -f 1). Finally, the calculated read count was converted to RPKM (reads per kilobase of exon per million reads mapped) (Fig 2A). To perform hierarchical clustering on the heatmap, web-based heat mapping tool, Heatmapper was employed (http://www.heatmapper.ca) [28]. Volcano plot was drawn using the R-package ggplot2 (ver 3.1.2).

### Identification of miRNA responsive elements (MREs)

To predict miRNAs-recognition elements capable of binding to LINEs and LTRs, which have significantly changed expression in tumors compared to normal, first, miRNA information in the mature form was downloaded from miRbase (release 22.1; https://www.mirbase.org/). Dog (canis familiaris; cf) and human miRNAs were selected from the downloaded data, and the sequences were extracted using the getfasta function of Bedtools with options (-s -name). Sequences of selected up- or down-regulated LINEs and LTRs were also extracted in the same way. By applying the miRNA-binding rule described by David P. Bartel (2019) [29], a python script for miRNA-binding prediction on expressed repetitive sequences into a total of 3 binding types (8mer, 7mer-m8, and 7mer-A1) was prepared and used in the analysis [30].

### Bioinformatic functional analysis

Gene ontology enrichment and pathway analysis was performed using Enrichr, a web-based comprehensive gene set enrichment analysis (https://amp.pharm.mssm.edu/Enrichr/) [31]. The oncoscore was calculated by the R package "OncoScore" to indicate the association between the genes and cancers based on PubMed citation [32].

### Cell culture

Breast cancer cell lines were purchased from Korean Cell Line Bank (Seoul, South Korea). MCF-10A was cultured with mammary epithelial growth medium supplements in mammary epithelial cell basal medium (Lonza, Basel, Switezerland). Roswell Park Memorial Institute 1640 medium (Hyclone, UT, USA) was used for SKBR-3, and Dulbecco’s Modified Eagle Medium/High glucose medium (Hyclone, UT, USA) was used for MCF-7 and MDA-MB-231. Cultures were performed at 37°C in a 5% CO2 and 10% fetal bovine serum (Hyclone, UT, USA) and 1% antibiotic-antimycotic (Gibco, MA, USA) were added to all culture media.

### Plasmid construction and transfection

The LINE sequence was inserted into the pCMV3 vector using KpnI and XbaI enzymes (Thermo Fisher Scientific, MA, USA). ENPP5 3’UTR sequence was amplified with MCF-10A genomic DNA, digested with AsiSi and XhoI enzyme (NEB, MA, USA), and inserted into pEZX-GA02 vector (GeneCopoeia, MD, USA). Lipofectamine 3000 (Thermo Fisher Scientific, MA, USA) for plasmid DNA and Lipofetamine imax (Thermo Fisher Scientific, MA, USA) for miR-126-5p mimic (Genepharama, Suzhou, China) were used for transfection following manufacturer’s instructions.

### RNA isolation and quantitative RT-PCR

RNA was isolated with Trizol (Invitrogen, MA, USA). Reverse transcription was performed with Omniscript RT Kit (Qiagen, Hilden, Germany) and CellScript All-in-One 5X First Strand cDNA Synthesis Master Mix (CellSafe, Gyeonggi-do, South Korea) using 1ug of Total RNA. For quantitative RT-PCR, all reactions were carried out in triplicate using SYBR green (Invitrogen, MA, USA), and the following primers were used: ENPP5-Forward (5’-AGTTTTGGGAAGAAGCGACAC-3’), ENPP5-Reverse (5’-GGCATGTAATGAGTAGGAAAGCG-3’), GNB2L1-Forward (5’-GAGTGTGGCCTTCTCCTCTG-3’), GNB2L1-Reverse (5’-GCTTGCAGTTAGCCAGGTTC-3’). For quantification of miR-126-5p, the stem-loop RT-qPCR Primers for miRNAs were designed using the web-based designing tool [33] and synthesized (Bioneer, Daejeon, Korea). Stem-loop RT primer was used for reverse transcription, and quantitative RT-PCR was performed using a pair of Forward (5’-GTTGGGGCATTATTACTTTTGG-3’) primer and a universal primer. The result was calculated by the 2-ΔΔ method. The GNB2L1 gene was used as an internal reference.

### Dual-luciferase reporter assay

MDA-MB-231 cells were seeded 24 h before transfection, and 10 pmol of miR-126-5p mimic, ENPP5-3’UTR vector, and LINE plasmid were co-transfected. Dual luciferase assay was performed using Secrete-Pair Dual Luminescence Assay Kit (GeneCopoeia, MD, USA) following the manufacturer’s instructions. Secreted Gaussia luciferase (GLuc) and secreted Alkaline Phosphatase (SEAP) reporter luciferase were measured, and luciferase activity was normalized by the ratio of GLuc to SEAP.

### Statistical analysis

All statistical analyzes were performed through Graph Pad Prism 8.0 software, and data were expressed as mean ± standard error of the mean (SEM). Statistical significance was evaluated by unpaired Student’s t-test, one-way ANOVA, two-way ANOVA. p-value < 0.05 was considered.

## Results

### Intact REs in human genome harbor various tumor suppressive miRNA responsive elements (MREs) sequences

**Fig 1:**
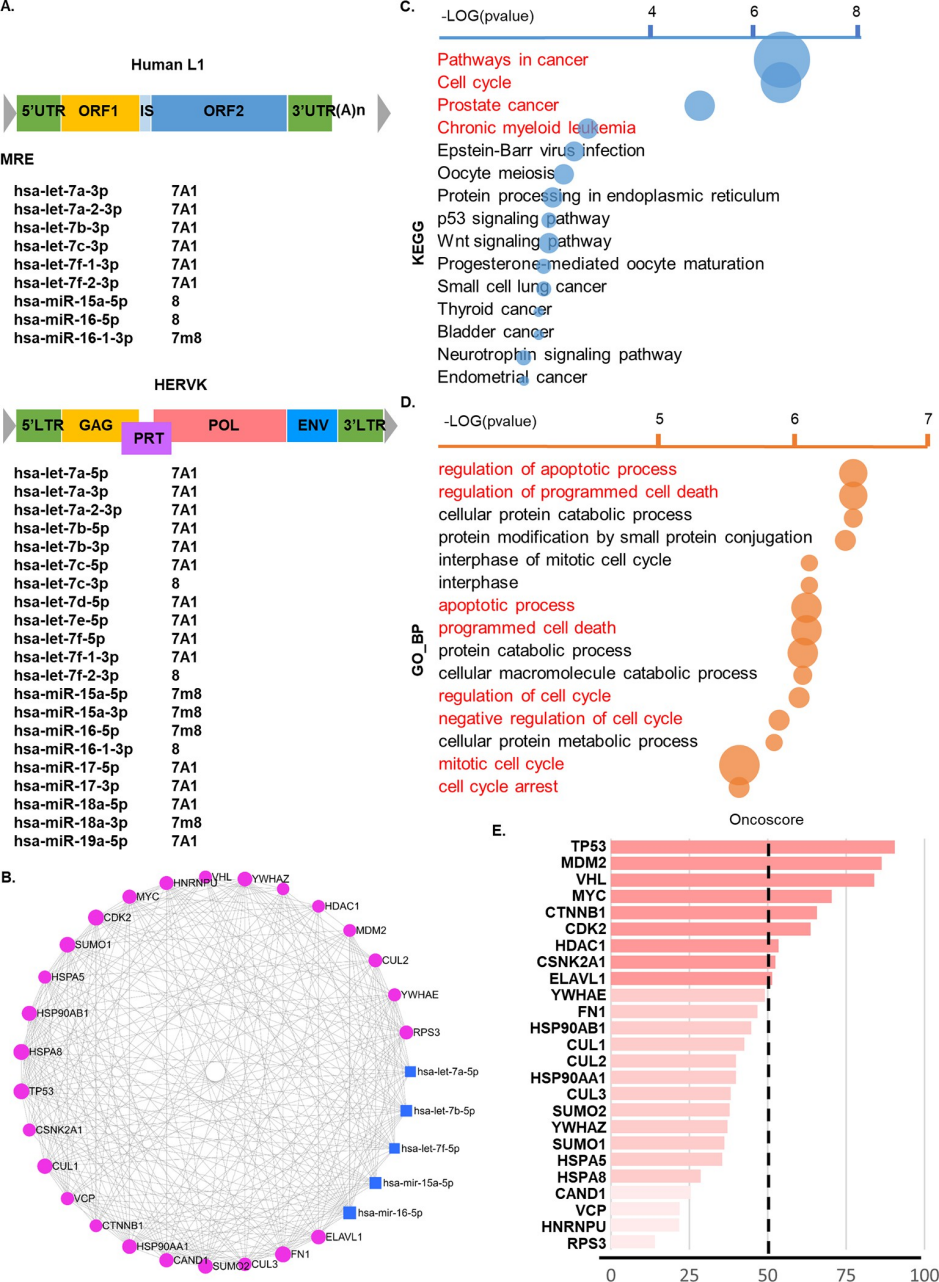
REs harbor numbers of miRNAs associated with tumor progression. **(A)** the list of miRNAs matched with MREs on the representative RE sequences. Top: LINE1, bottom: HERV-K. **(B)** miRNA and gene network analysis based on the RE-derived MREs. **(C)** KEGG pathway analysis. Circle size represents numbers of genes; x-axis in -log (p-value). **(D)** Gene ontology (GO) analysis was performed to identify those biological process, **(E)** The Oncoscore was measured for 25 major target genes responsible to the miRNAs identified.

REs-related sequences, which account for more than 40% of the human genome, are mostly defective forms that have lost their functions [34]. We have confirmed which MREs are exist in the full-length RE sequence known as the provider. Among the sequences known LINE1 and human endogenous retroviruses, HERV-K109 (AF164615) and LINE1 (MZ092700) were used as reference RE sequences that fully retained their structure. As a result, several MREs such as the MREs of the let-7 and miR-15/16 clusters known as the typical cancer suppressor miRNA were found from the two RE reference sequences (Fig 1A). Network analysis using the MREs-corresponding miRNAs showed that they were directly related to the regulation of various cancer-related genes such as TP53 and MYC (Fig 1B). Pathway and gene enrichment analysis for more detailed functions of the related genes revealed that the term of “pathways in cancer” is the most frequently found, followed by “cell cycle”, “prostate cancer”, and “chronic myeloid leukemia associated pathway” (Fig 1C). Gene Ontology (GO) analysis identified the apoptotic process, cell death, and cell cycle-related biological process (Fig 1D). Molecular function related to protein binding such as enzyme binding and transcription factor binding was identified, and several terms of protein and macromolecular complex and cellular organelles were found in the cellular components (S1A and S1B Fig). These network analyses of MREs-corresponding miRNA target genes indicate that RE expression is capable of associating with diverse biological roles. Furthermore, many genes whose expression may link to RE expressions through the RE-derived MREs were associated with oncogenesis (Fig 1E). The Oncoscore analysis showed high scores for the target genes such as TP53, MDM2, VHL, MYC, CTNNB1, CDK2, HDAC1, CSNK2A1, and ELAV1, frequently reported in cancer-related studies (Fig 1E). Further question then arose how these gene expression associates with REs. Various epigenetic regulation mechanisms, such as methylation and histone modification, have been known in the suppression of RE expression. On the other hand, the role of the Apobec family in antiretroviral and retroelements is a well-known genetic mechanism leading to the physical blocking of reverse transcription. We further analyzed how the genes playing an important role in the development of cancers have an association with Apobec3c, known to inhibit RE expression in cells. Interestingly, many of these genes showed a negative association with APOBEC3C expression (S1C Fig). Altogether, aberrant RE expression is capable of influence on the regulatory action of cancer-suppressing miRNAs in cells.

### RE expression robustly increased in HBC transcriptome data set

**Fig 2:**
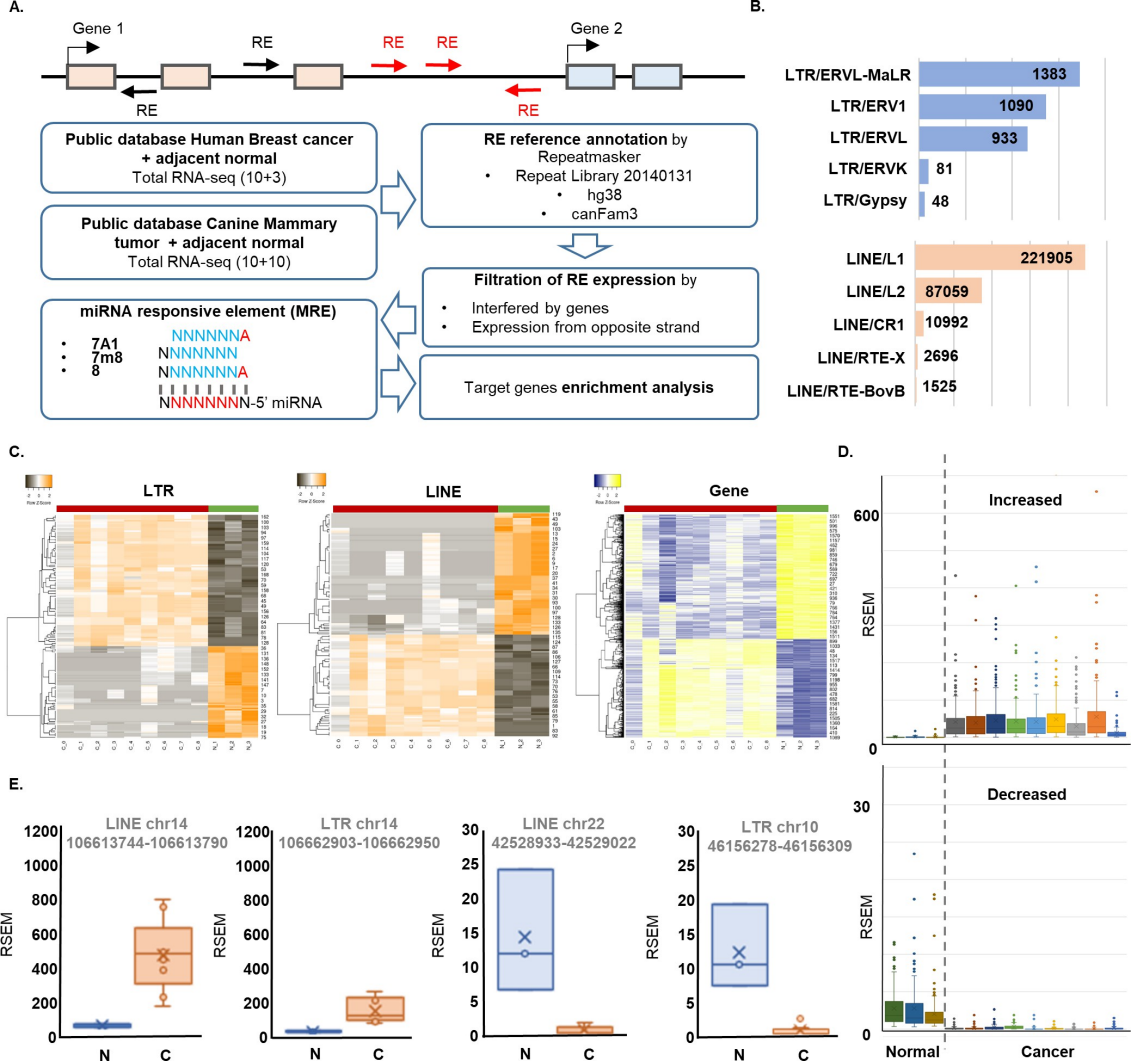
REs expression in the transcriptome data set of HBC and normal. **(A)** Scheme of REs annotation followed by MRE identification. Only intergenic REs expression was considered for further analysis. **(B)** Numbers of REs identified. Five major subfamilies from each LTR and LINE group were listed, respectively. **(C)** Heatmap clustering using differentially expressed REs (LTR, LINE) and genes. **(D)** Box and Whisker plot of increased and decreased RE in normal and cancer samples. **(E)** LINE and LTR loci significantly up- and down-regulated in HBC.

Recently, many RNA-seq data have been analyzed from HBC patients and normal samples, accumulated, and used in the public database. We secured RNA-seq data of three normal and 10 HBC tissues listed in NCBI and annotated the expression and gene expression of LINEs and HERVs through the Repeatmasker. To prevent interference from gene-related transcripts, this study analyzed transcripts only from intergenic positions that are distinct from genes. MREs were identified from the sequences of differentially expressed REs for eight seed bases known to be essential for miRNA binding [35]. Putative target genes and their functions were identified from the MREs-corresponding miRNA for the enrichment analysis. Anti-sense, which exists inside the gene or is expressed opposite to the gene, was excluded in this study due to its functions directly associated with genes (Fig 2A).

As a result, MREs for the MaLR was identified as the highest frequency in the LTR group, followed by ERV1 and ERVL. In the LINE group, L1 showed an unrivaled high frequency, followed by L2 and CR1 (Fig 2B). Heatmap clustering analysis shows the significantly distinguished expressions of genes and REs (HERVs and LINEs) clearly distinguished between cancer and normal (Fig 2C). Box and whisker plot presenting mean RSEM values showed dramatical changes of REs expression between normal and HBC samples (Fig 2D). Increase level is more than 30 times than the decrease level in cancers (Fig 2D). The box plot presents representative individual RE expression at four locations in the normal and cancer groups. LINE (chr 14:106613744) showed an increase in mean expression by about 500 times or more in cancer. In addition, in the case of the LTR (chr14:106662903) the expression was increased in HBCs by more than 100 times. Conversely, in the case of LINE (chr22:42528933) and LTR (chr10:46156278) the expression which were weak in normal group (less than 25 RSEM) has been depleted (Fig 2E). These results confirm that genome-wide RE expressions are robustly increased in HBC.

### Diverse MREs were identified from the differentially expressed REs in HBC

**Fig 3:**
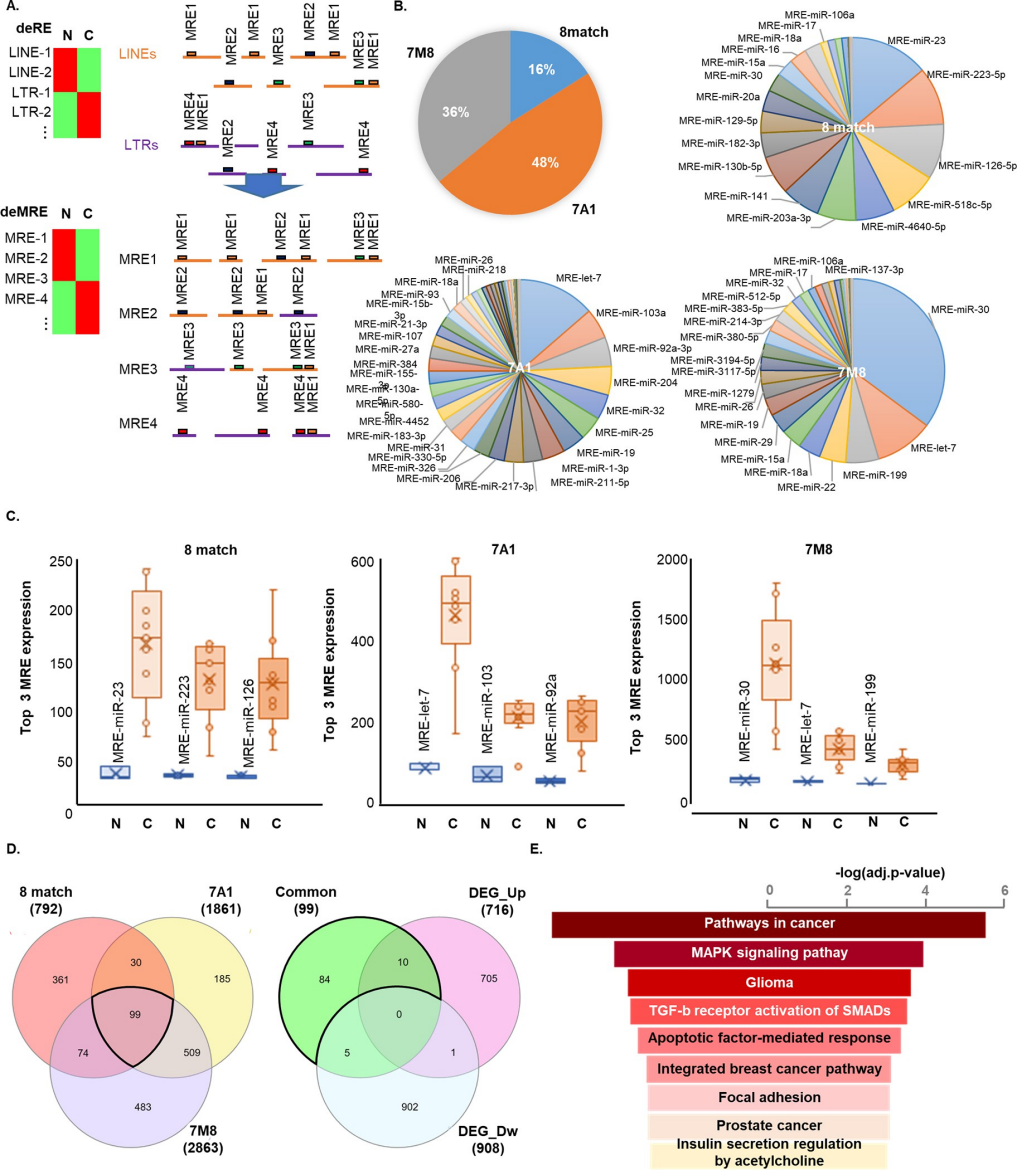
MREs quantification from REs expression. **(A)** Conceptual MREs identification and quantification. Differential expression of REs is converted into the differential expression of MREs. **(B)** Left: MREs were sub-classified according to the seed matching patterns (8M, 7A1 and 7M8, See the main text for definition). Right 3 circle graphs: Proportions of the MREs corresponding miRNAs in each seed matching pattern. **(C)** Box and Whisker plot of significantly increased MREs in cancer. Top 3 MREs in each seed matching patterns were presented in each graph. **(D)** The list of putative target genes in each group, 8M, 7A1 and 7M8 was compared to define common target genes. Venn diagrams shows 99 common target genes among three 8M, 7A1 and 7M8 groups (left). The list of putative target genes which are expected to be down-regulated was compared with the list of differentially expressed (up- or down-) genes (DEGs) in transcriptome data set to see influence by MREs (Right). The 94 out of 99 were not down-regulated in HBC. **(E)** Gene enrichment analysis using the 94 putative target genes showed that the genes are involved in the term of pathways in cancer most.

MREs was defined from the sequence of the RE transcripts increased in HBC (Fig 3A). Since types of seed match can have different efficiency [36], we identified MREs in three categories; 1) 8M MREs which all eight nucleotides are matched with miRNA’s seed sequence; 2) 7A1 MREs which seven nucleotides are matched but one nucleotide is mismatched in the first base; 3) 7M8 MREs which seven nucleotides are matched but one nucleotide is mismatched in the 8th base. A total MREs are composed of 16% 8M, 48% 7A1 and 36% 7M8.

MRE-miR-23, MRE-miR-223-5p, and MRE-miR-126-5p were most frequently found in the 8M. MRE-let-7, MRE-miR-103a, and MRE-miR-92a-3p are frequent in the 7A1 group. In the 7M8 group, the MRE for miR-30 was significantly high, followed by hsa-let-7 and hsa-miR-199. The miRNA Let-7, the most frequently found from the reference LINE and HERV sequences were identified in both 7A1 and 7M8 groups. It is an impressive finding because, except miR-23, which has been studied to have both tumor-suppressive and promoting functions, the other miRNAs hsa-miR-223-5p and hsa-miR-126-5p have been known as tumor-suppressive miRNAs in the 8M group [37, 38]. Moreover, let-7 is known as a representative tumor-suppressive miRNA [39]. Hsa-miR-30 and hsa-miR-199, which were the most frequent in the 7M8 group, have also been reported as tumor suppressive functions (Fig 3B) [40, 41]. In the 7A1 group, however, has-miR-103a and has-miR-93a have more frequently been reported as tumor-promoting miRNAs. Using the catalog of MREs, the REs expression data was converted into MREs to measure MRE expression levels. The expression of top 3 MREs in each group are depicted in the box and whisker plots. All are drastically increased in HBC. Among them, MRE-miR-30 in the 7M8 group showed the highest increase (Fig 3C). We hypothesized that the MREs would interfere with the regulation of miRNAs on the downstream genes and thus screened the genes commonly affected by these three groups of MREs. Ninety-nine putative target genes were identified and compared with the differentially expressed genes (DEGs) between normal and HBC. By excluding down-regulated DEGs, ninety-four genes were finally considered as the genes being out of miRNA controls (Fig 3D). The gene enrichment analysis was most associated with cancer-related pathways and cell activity-related MAPK signaling pathways (Fig 3E). These results made it possible to predict that the increased RE transcripts in HBC can play oncogenic roles in association with miRNA function.

### MRE-miR-126 was commonly found from the up-regulated REs in CMT and HBC

**Fig 4:**
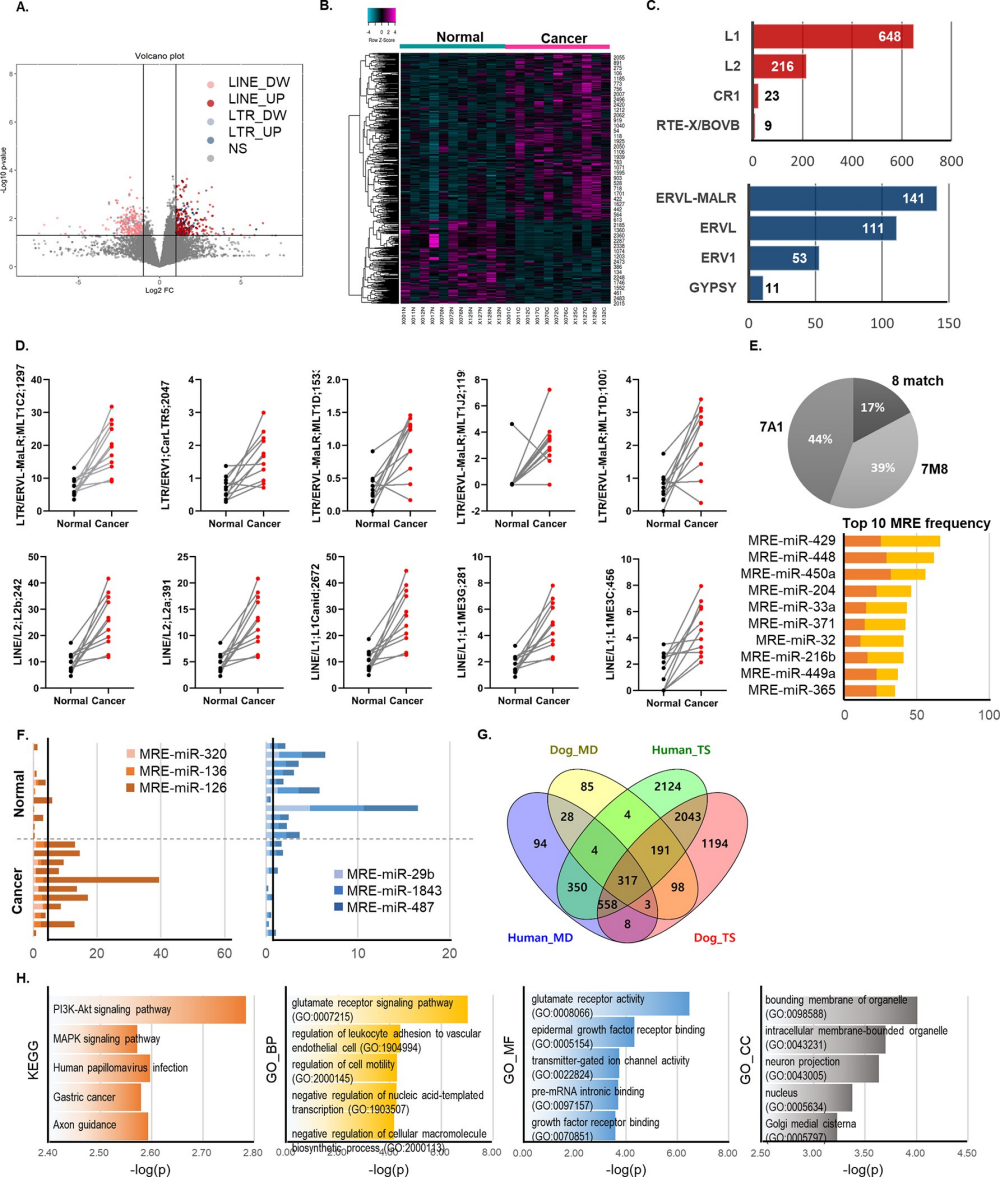
REs expression and MREs identification in the transcriptome data set of CMT. **(A)** Volcano plot presented up- and down-regulated REs in the RNA-seq analysis of CMT tissues. Lines indicate significant levels p-value<0.05 and |foldchange|>2. **(B)** Heatmap clustering using differentially expressed REs. Up-regulated REs are more numbers than down-regulated REs. **(C)** Four major subfamilies from each LTR and LINE group respectively were listed for those differentially expression REs. **(D)** Ten representative RE loci significantly up-regulated (LTR, LINE) in paired normal and cancer samples. **(E)** MREs found in the three categories (8M, 7A1 and 7M8), and top 10 MREs frequently found in LINEs and LTRs. **(F)** The expressions of top three MREs on the differentially increased and decreased REs in the normal and tumor tissues. **(G)** Venn diagram showing 317 miR-126 target genes commonly identified in human and dog databases. MD represents miRDB database and TS represents Targetscan database. **(H)** Top five terms in the KEGG pathway and GO analyses of biological process (BP), molecular function (MF) and cellular component (CC) using the 317 miR-126 target genes.

The similarities between HBC and CMT are known in many aspects. We confirmed from these two cancer transcriptome data whether the miRNA regulatory interference by RE-derived MREs was similar between different species. To identify the significantly increased REs in CMT, we also quantified the RE expression in our RNA-seq data [42] (S2 Fig). RE annotation was performed in the same order as HBC transcriptome analysis, and approximately 1.5 million RE loci are defined by Repeatmasker canine database. Transcripts related to cfLINEs and cfERVs were identified in a wide range of sizes. Intergenic REs expression in dog genome was detected in 922,470 loci consisting of 681,292 cfLINEs and 241,178 cfERVs. After filtration by size and expression level, a total of 34,633 transcripts were processed further to identify differential RE expressions. The volcanic plot clearly showed an increase or decrease in RE in CMT (Fig 4A), and a total of 1,212 REs was identified as differentially expressed REs (p-value <0.05, |log2foldchange|>1). Differentially expressed REs between individual samples and chromosomes are depicted in heatmap clustering (Fig 4B). In the subtype distribution of differentially expressed REs, ERVL-MALR (45%) was the most common followed by ERVL (35%) and ERV1 (17%) in the long terminal repeat (LTR) group which includes gypsy, copia, BEL/pao and ERVs. In LINE group, L1 (72%) and L2 (24%) occupied most of the REs (Fig 4C). Ten representative RE expressions in normal and CMT showed individual REs expression was significantly increased in CMT (Fig 4D). When MRE was identified from the RE transcripts differentially expressed in CMT, 8M MREs accounted for 17%, 7A1 MREs for 44%, and 7M8 MREs for 39%. It is similar to those shown in 16%, 48%, and 36% of humans, respectively (Fig 4E).

The highest increased-MREs in CMT were responsible for cfa-miR-320, cfa-miR-136, and cfa-mir-126, while the decreased MREs were against miRNAs of cfa-miR-29b, cfa-miR-3843, and cfa-miR-487. Interestingly, the three highest increased MREs, are responsible to cfa-miR-320, cfa-miR-136, and cfa-miR-126 that are all reported cancer-suppressive functions. Conversely, decreased MREs were responsible for oncogenic miRNAs, cfa-miR-29b and cfa-miR-487a, except for miR-1843 which has not studied much (Fig 4F) [43–47]. We also investigated the putative functions of these RE transcripts as a provider of MREs by looking at the target genes of miRNAs. Enrichment analysis was performed using the list of putative target genes of miR-126 as a representative miRNA commonly found in both HBC and CMT. A total of 317 genes shared by two different miRNA target search algorithms, miRDB and Targetscan, which also provide dog database, were identified as target genes of miR-126 (Fig 4G) [29, 48]. The increased MREs may disturb corresponding miRNAs regulation on target genes resulting in increased target gene expressions. KEGG pathway enrichment analysis revealed that the list of genes was associated with PI3K-Akt signaling pathway and MAPK signaling pathway which are well known in cancers. One interesting finding was that glutamate receptor signaling pathway and activity were significantly enriched in GO database (Fig 4H). Of note, aberrant glutamate signaling has been recently shown to play a role in the transformation and maintenance of various cancers [49]. Through these analyses, we confirmed that the disruption of tumor suppressive miRNAs by REs overexpression is also conserved in CMT as well as in HBC.

### REs expression has a crucial role in HBC and CMTs as competing endogenous RNAs for tumor-suppressive miRNAs

**Fig 5:**
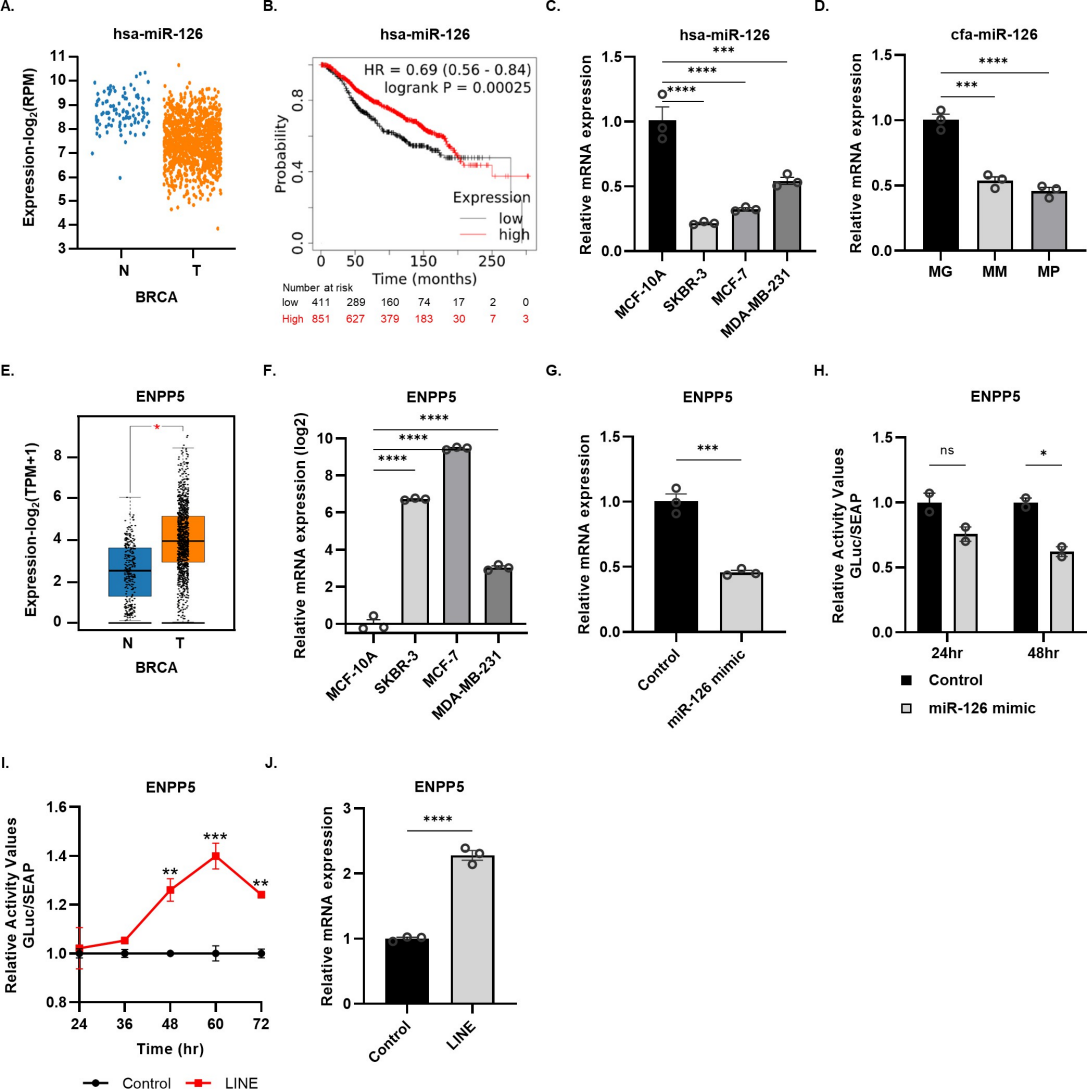
Validation demonstrating the role of LINE1/miR-126/ENPP5 axis. **(A)** Hsa-miR-126 levels in BRCA public database. **(B)** Hsa-miR-126-5p expression in HBC cell lines compared to normal MCF-10A cells. **(C)** Kaplan-Meier plot showed that highly expressed hsa-miR-126 associated with better overall survival in HBC patients. **(D)** cfa-miR-126 expression was down-regulated in CMT cell lines when compared with normal mammary gland. **(E)** ENPP5 expression was significantly up regulated in BRCA public database. **(F)** ENPP5 expression was also significantly up-regulated in HBC related cell lines of SKBR-3, MCF-7 and MDA-MB-231. Log2-transformed expression was used **(G)** miR-126-5p (mimic) transfection significantly reduced the ENPP5 mRNA levels. **(H)** miR-126-5p transfection (mimic) also significantly reduced the luciferase activity conjugated with the 3’UTR of ENPP5. **(I)** The luciferase activity conjugated with the 3’UTR of ENPP5 was significantly increased by the overexpression of LINE in MDA-MB-231 cells. **(J)** ENPP5 mRNA was significantly increased by LINE overexpression in MCF-10A cells. *, **, *** and **** indicate significance p<0.05, 0.01, 0.001, and 0.0001, respectively.

Through the bioinformatic analysis, we demonstrated a common increase in RE-derived MREs for miR-126 in HBC and CMT samples. We thus confirmed whether the overexpression of REs could affect the regulatory functions of miR-126. The expression of miR-126 decreases in cancer in the BRCA transcriptome database (Fig 5A). On the contrary, the expression of high miR-126 in cancer patients showed better overall survival (OS), showing the anti-cancer role of miR-126 (Fig 5B). These characteristics can also be seen in the cell lines of mammary gland cancer and breast cancer in humans and dogs. The expression of miR-126 was significantly lower in both cancer cell lines than in normal cell lines and tissues (Fig 5C and 5D). We identified ENPP5 as a target gene of hsa-miR-126-5p using the miRDB and the TargetScan human databases. ENPP5 mRNA expression was increased in human breast cancer patient samples (Fig 5E), and significantly higher in human breast cancer cell lines compared to normal human breast cell line MCF-10A (Fig 5F). It indicates that ENPP5 is upregulated in breast cancer oppositely to miR-126. To confirm that ENPP5 is a target gene of hsa-miR-126-5p, we transfected hsa-miR-126-5p mimic to MDA-MB-231 cells and confirmed the ENPP5 mRNA level decrease (Fig 5G). The 3’ UTR of the ENPP5 gene (GRCh38: chr 6:46159210–46161229:-1) was tagged it onto the end of luciferase gene. The ENPP5 reporter was also significantly decreased by miR-126-5p mimic in the luciferase reporter assay (Fig 5H). The result indicates that ENPP5 is a direct target gene of hsa-miR-126-5p. Finally, we tested whether overexpression of LINE protects the expression of ENPP5 and thus the REs works as a ceRNA against hsa-miR-126-5p. The ENPP5 mRNA level was significantly increased by LINE overexpression in the luciferase system (Fig 5I). At various levels, these results were confirmed in other HBC cell lines (MCF-7 and SKBR-3) (S3 Fig). LINE overexpression in the MCF-10A, whose ENPP5 expression was low but has-miR-126-5p was high, significantly increased ENPP5 expression (Fig 5J).

## Discussion

Our current study is based on the findings of the ENCODE project demonstrating more than 80% of genome are transcribed to RNAs. Since REs comprised approximately 50% of human genome, it is expected that much of these transcripts come from REs. However, until now most transcriptome analysis has been conducted to focus on the genes which are composed of 1.2% of entire human genome. The RE transcriptome was rarely analyzed because of not only uselessness ‘called as junk’ but also the limitation of RE analysis using high-throughput sequencing with the short reads. More attentions onto REs and their functions in both normal and diseases condition are necessary for better understanding in genome molecular biology.

**Fig 6:**
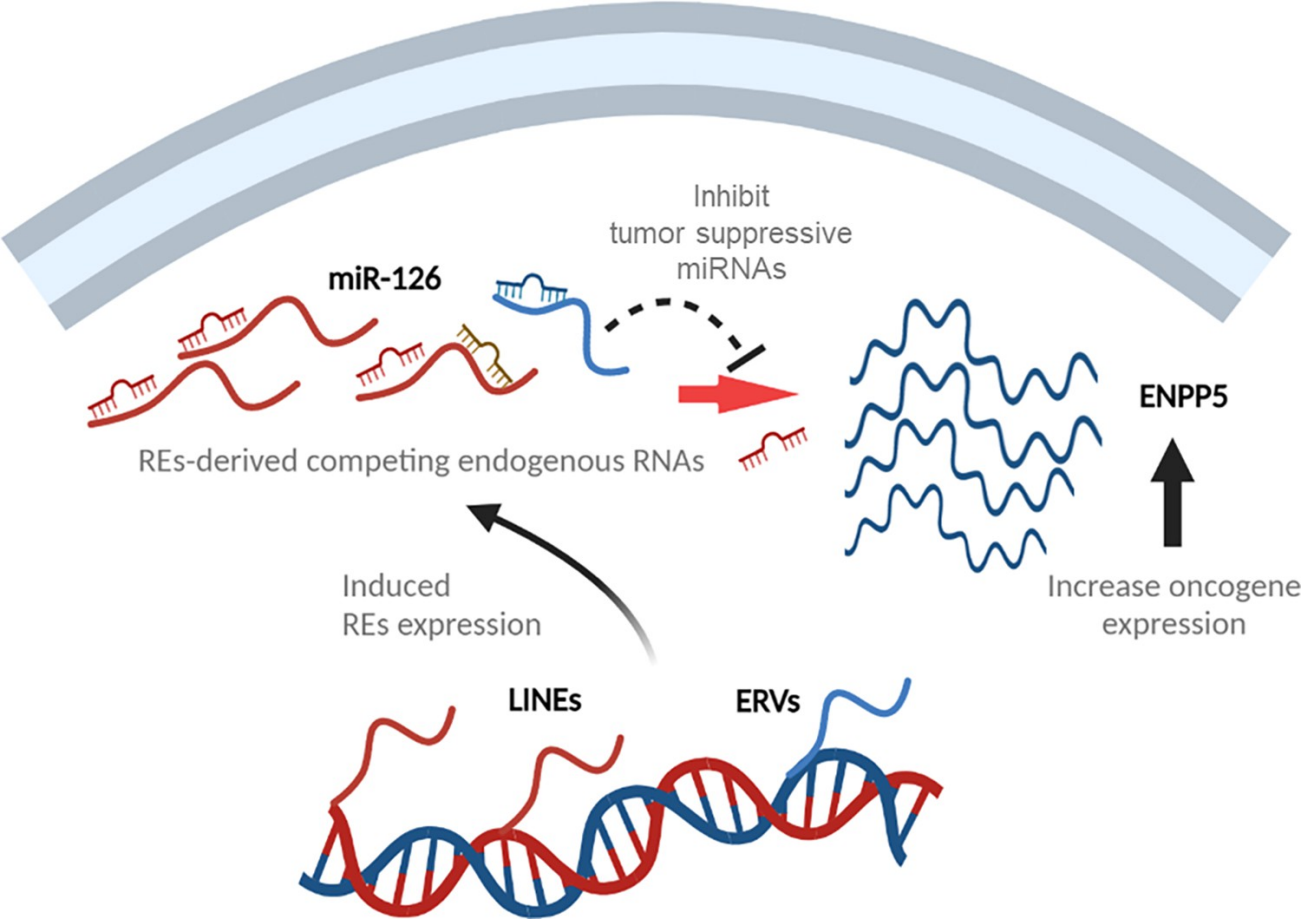
Putative role of RE transcripts competing with target genes of miR-126. LINEs and ERVs increased in tumorous condition can interfere tumor suppressive miRNA, such as miR-126 resulting in increased of oncogenic gene expression (ENPP5).

The association of RE expressions with cancerization has been reported, and their functions are suggested at various levels [50]. Most REs are in the form of partial and fragmented RE sequences and some of the REs expression increases in cancer conditions. Recently, competing endogenous RNA (ceRNA), a long non-coding RNA capable of binding to miRNAs and thus regulating the expression of target mRNAs, has been suggested [51, 52]. However, ceRNA regulation and its effects continue to be debated. In this study, we demonstrated that ceRNAs from REs act as a sponge for tumor suppressive miRNAs. Certain RE expressions with MREs were increased in HBC and CMT and thus gave tumor promoting effect. This study suggests that aberrant REs expression is the source of ceRNAs that influence the tumor suppressive function of miRNAs (Fig 6).

Unfortunately, this study has a limitation that we only investigated the MREs on the significantly increased REs in cancer. The MRE investigation in the overall REs expression will provide better information to understand the role of RE as a ceRNA. The total amount of individual MREs is more meaningful than the source of particular RE types. For instance, differentially expressed (increased or decreased) REs could share MREs that compensate for MRE changes. However, this study is still worthwhile because most of the REs we identified are significantly increased in cancers.

CMT is used as an animal model due to its high similarity to HBC. We thought that the roles of REs might be specific to each species depending on the type and sequence of REs, but the evolutionarily conserved REs and their functions would be significant. It suggests that the expression of REs can be involved in the regulation of miRNAs in humans and other mammals at least in disease conditions including cancers. As an example of the tumor promoting function of MREs, we found that MRE-miR-126 is commonly elevated in HBC and CMT. The miR-126 is a tumor suppressor and its expression is reduced in HBC. It is known that the treatment of miR-126 inhibited cancer cell proliferation and induced G1 arrest [53]. This study showed that the L1 expression could influence the role of miR-126. Overexpression of L1 sequence in cancer blocked the depletion of ENPP5, a target gene of miR-126. It was confirmed through luciferase analysis that miR-126 suppressed the expression of luciferase conjugated with the 3’-UTR of ENPP5, and L1-expression released the luciferase expression from the miR-126 regulation. The regulation of miRNA-LINE-ENPP5 was also tested in the MCF-7 and SKBR-3 breast cancer cell lines (S3 Fig). Similar regulation was observed in MCF-7, but not in SKBR-3. LINE overexpression in SKBR-3 did not protect ENPP5 from miR-126, resulting in a non-significant increase in ENPP5 levels. The major difference between SKBR-3 and the other cell lines was HER2 status. Only SKBR-3 was HER2+. Further studies are needed to determine if HER2 signaling regulates ENPP5. This is an interesting area of research, as ENPP5 has recently been proposed as a driver of triple-negative breast cancer, a major enzyme of the lysophosphatidic acid pathway [54].

ENPP5 expression levels in the cell line, MDA-MB-231 about five times higher than in MCF-10A. This study provides critical clues that the increased expression of miRNA responsive elements in retroelements across the genome can play a crucial role in tumorigenesis by regulating tumor suppressive miRNAs and thus relieving the blockade of oncogene expression.

## Supporting information

S1 Fig Two other categories of GO analysis **(A)** molecular functions and **(B)** cellular compartments using the putative target genes of miRNAs identified by the MREs on the reference RE sequences **(C)** The Pearson correlation between the expression of putative target genes with APOBEC3C gene expression in public database. ND: not determined.
(TIF)

S2 Fig RE expression was measured from CMT transcriptome data. The procedure, parameters and intermediated results were summarized.
(TIF)

S3 Fig Validation of the association of LINE with miR-126 on the ENPP5 expression **(A)** miR-126 (mimic) successfully reduced ENPP5 mRNA level in other breast cancer cell lines MCF-7 and SKBR-3. **(B)** LINE overexpression increased ENPP5 mRNA level in MCF-7 cells but not in SKBR-3. *, ** and *** indicate significance p<0.05, 0.01, and 0.001, respectively. ns means not significant.
(TIF)

## Abbreviation

RE: retroelement
HBC: human breast cancer
CMT: canine mammary tumor
MRE: miRNA responsive element
ceRNA: competing endogenous RNA
LINE: long interspersed nuclear element
SINE: short interspersed nuclear element
HERV: human endogenous retrovirus
LTR: long terminal repeat
ERV: endogenous retrovirus
Cf: canis familiaris
ncRNA: non-coding RNAs
miRNA: microRNA
ORF: open reading frame
OS: overall survival
