# Supplementary figures and images for: New oncogenic functions of LINE1 retroelement as a ceRNA for tumor suppressive microRNA miR-126 on ENPP5
Source: PLoS One. 2023 Jun 23;18(6):e0286814. doi: 10.1371/journal.pone.0286814 (PMC10289412; doi:10.1371/journal.pone.0286814)

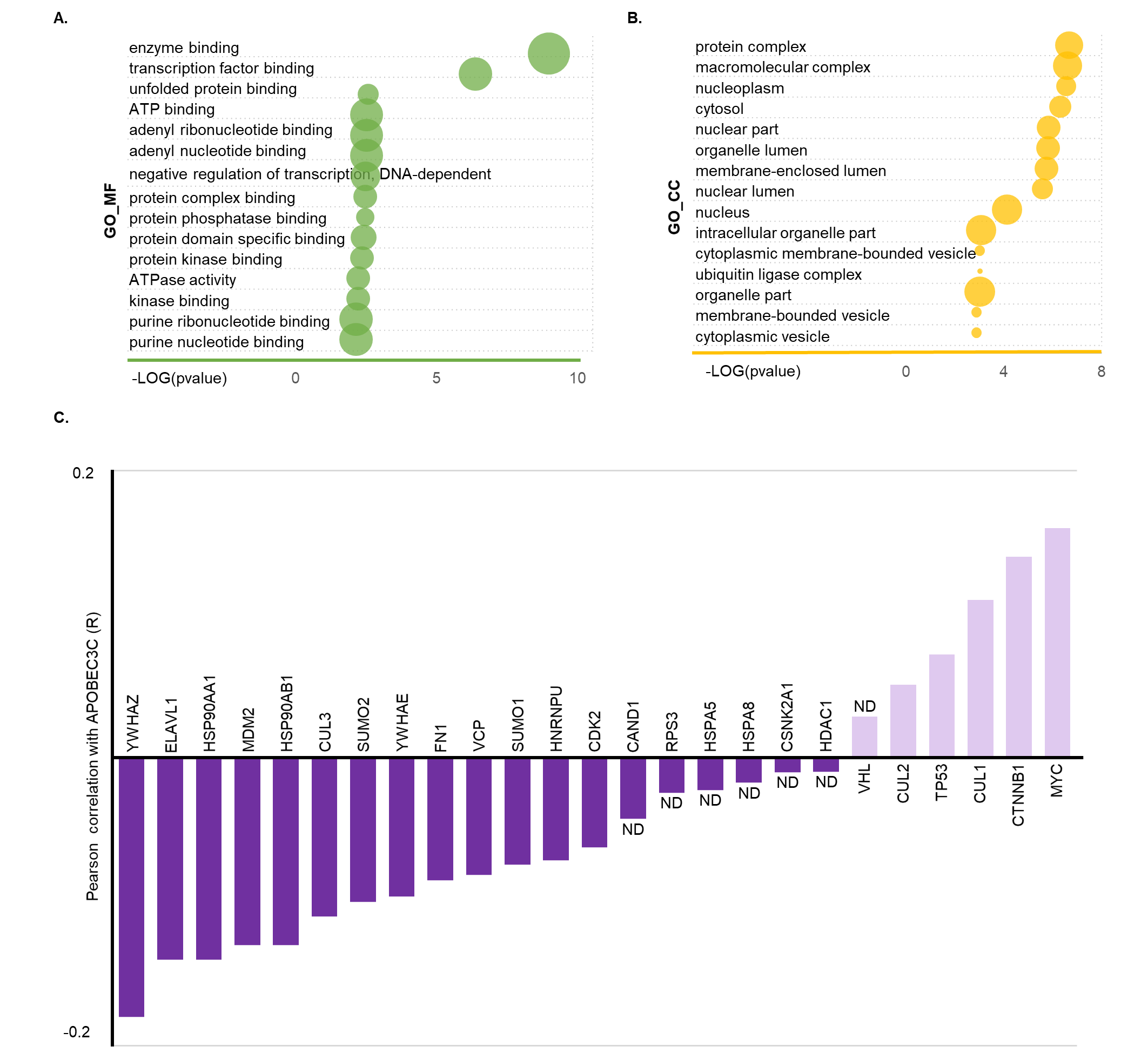

Supplement: S1 Fig — Two other categories of GO analysis (A) molecular functions and (B) cellular compartments using the putative target genes of miRNAs identified by the MREs on the reference RE sequences (C) The Pearson correlation between the expression of putative target genes with APOBEC3C gene expression in public database. ND: not determined. (TIF) [file pone.0286814.s001.tif]

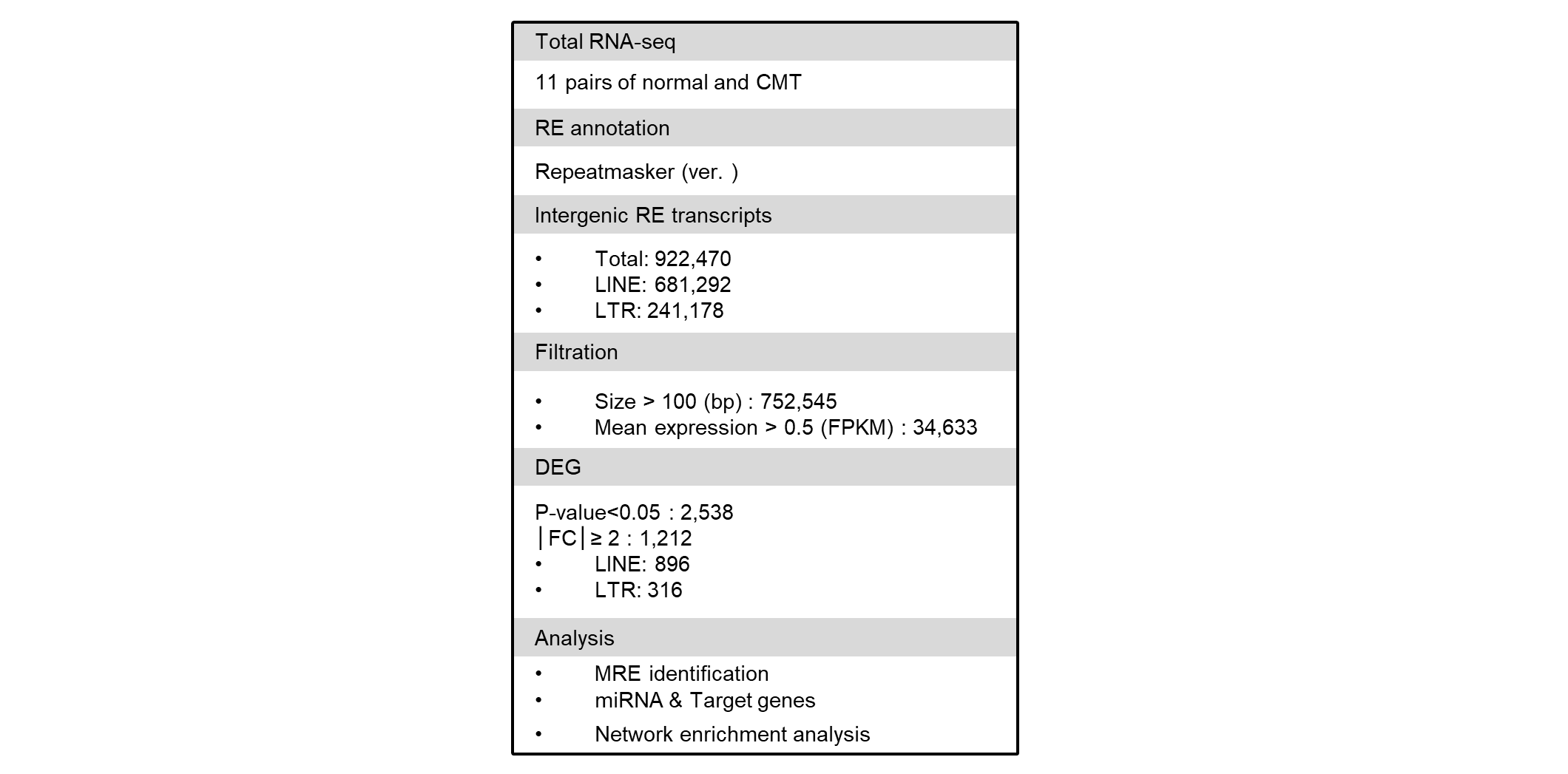

Supplement: S2 Fig — The procedure, parameters and intermediated results were summarized. (TIF) [file pone.0286814.s002.tif]

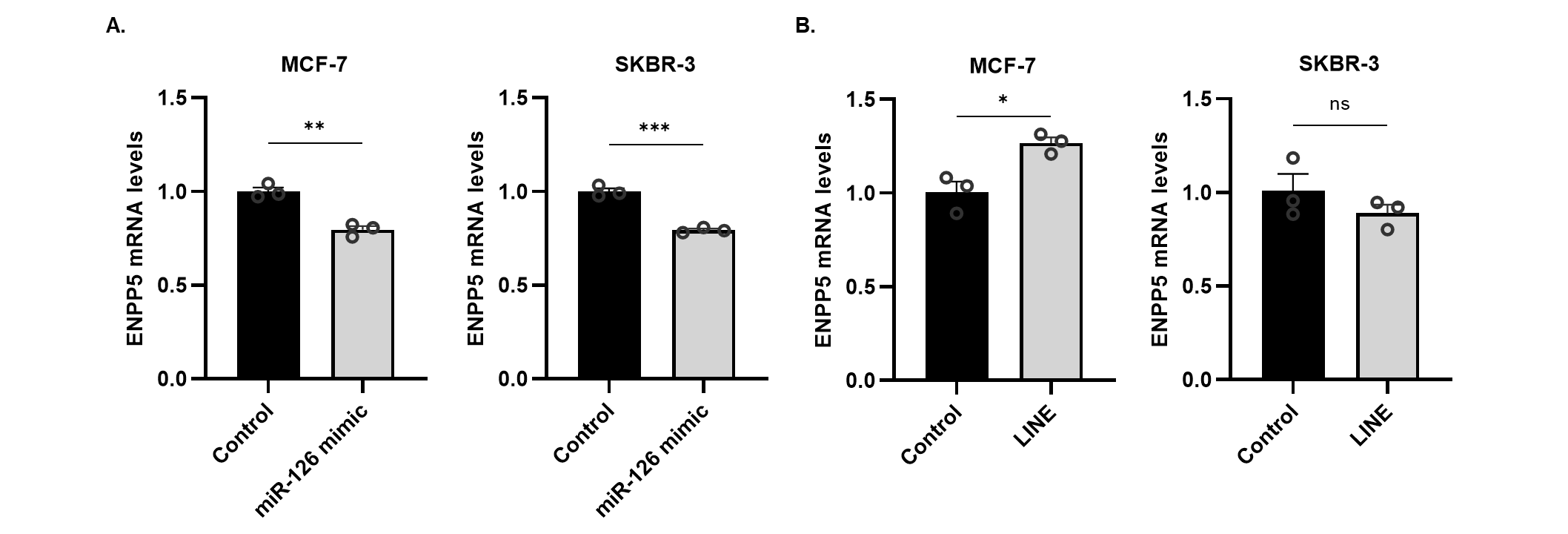

Supplement: S3 Fig — Validation of the association of LINE with miR-126 on the ENPP5 expression (A) miR-126 (mimic) successfully reduced ENPP5 mRNA level in other breast cancer cell lines MCF-7 and SKBR-3. (B) LINE overexpression increased ENPP5 mRNA level in MCF-7 cells but not in SKBR-3. *, ** and *** indicate significance p<0.05, 0.01, and 0.001, respectively. ns means not significant. (TIF) [file pone.0286814.s003.tif]
